# Improving Participant Adherence in Clinical Research of Traditional Chinese Medicine

**DOI:** 10.1155/2014/376058

**Published:** 2014-01-09

**Authors:** Wenke Zheng, Bai Chang, Jing Chen

**Affiliations:** ^1^Tianjin University of Traditional Chinese Medicine, Tianjin 300193, China; ^2^Center for Evidence Based Medicine, Tianjin University of TCM, Tianjin 300193, China; ^3^Metabolic Disease Hospital, Tianjin Medical University, Tianjin 300070, China

## Abstract

Ensuring good participant adherence in clinical trials plays an important role in that poor adherence may jeopardize the internal validity of the trial. Improving adherence in clinical trials on traditional Chinese medicine (TCM) has long been a concern for Chinese researchers who are conducting clinical trials. Drawing on from our past experiences in managing patient adherence in large-scale clinical trials, we identified factors that influence adherence and categorized them by sources into factors with respect to the trial protocol, on the part of the patients and the investigators. On this basis, we developed a series of ways to improve participants' adherence, while taking into account the characteristics of TCM trials, in the hope of providing reference for peer clinical researchers.

## 1. Introduction

Participants' adherence can be defined to describe the implementation of prescribed medical orders in clinical trials. It refers to the subjects' acceptance of prescribed treatment which contained the dosages and courses and followup. Adherence is closely related to the quality of clinical trials; low patient adherence with prescribed treatments is a very common problem in clinical trials and can seriously distort the generalizability and validity of controlled clinical trials [1]. A research showed that different adherence can lead to difference of end events significantly [2]. In addition, it can also save funding and reduce cycle time when improving the adherence in clinical trials.

The adherence in clinical trials had been drawing more and more attentions of researchers abroad in the 1990s, and “The Journal of Compliance in Health Care” pays special attention to it. The phenomenon of non adherence is widespread among clinical trials [1, 3]. A study involved 1367 participants with hypertension showed that there are only 15.9% high adherers [4]. Some patients take medicine by an act of volition, and in double-blind, placebo-controlled trials, the code that was broken can lead to poor adherence also [5].

Poor adherence includes two aspects: one is adherence of drug therapy; that is, the patients fail to take drug in accordance with the prescribed methods of taking medicine, or accept the forbidden treatment, another one is adherence with followup; that is, patients do not follow up or get check on schedule or drop out without any reason during the trial.

For the particularity of TCM clinical trials, there are some characteristic in patients adherence; part of the western medicine doctors mistrust the curative effect of TCM; their negative attitudes toward the TCM trials can lead to the poor adherence. The long course of treatment for the chronic disease can impact the adherence, and some other factors such as the mega dose of TCM, take medicine frequently, the unpalatable of decoction also can hinder adherence. Moreover, it is difficult to produce Chinese medicine placebo, so in a long-term test, once the placebo is recognized, adherence of drug therapy will be influenced.

On the other hand, compared with Western medicine, there are less ADR and invasive test in TCM, and it can improve the adherence to some extent.

Through the implementation of large-scale multicenter clinical trial of TCM—“Qi Shen Yi Qi dropping pill for myocardial infarction's secondary prevention study” (MISPS-TCM) successfully, the nonadherence was observed from the main performance: some patients can not follow up or take medicine on time; some patients refused to return the surplus medicine for various reasons; some participants refused to continue to take drugs once any adverse event appeared, regardless of whether it was related to the drug or not; Some patients were lost for followup at some stages of the trial with unknown cause [6].

Through the MISPS-TCM, we established a quality management system of clinical trial. Improving the patients adherence is part of the system. According to the project implementation, we will have a discussion about the methods to improve patient's adherence.

## 2. Factors Related to Participant Adherence in Clinical Trials of TCM

There are many factors which can influence the subjects adherence, among the hypertensive patients with poor adherence, the factors contained forget, discontinue taking the antihypertensive medications as blood pressure has been controlled, unwilling to take medicine at all, fear of adverse reactions, control blood pressure by other ways, the cost, and so on [7]. Another study showed that [8] the poor taste of herbs, troublesomeness of boiling, and worrying about the quality of medicines also are factors of poor adherence.

Studies suggested that the adherence of long-term patients is lower than short-term [9] and adherence of patients with acute disease is better than chronic disease. And the adherence will markedly reduce after subjects have received six months treatment [10–12].

In the MISPS-TCM, there were 467 discontinuations, which can be known as a form of non-adherence. The discontinuations contained 142 for disobedience (30.41%), 130 drops with unknown reasons (27.84%), 82 for adverse events (17.56%), 58 for lack of efficacy (12.42%), 3 for the usage of the forbidden drugs (0.64%), and 52 for other reasons (11.13%). For the main purpose of the trial, we did not focused on the determinants of patient adherence; thus, there have been no more detailed data to analyse the factors. But the staff of MISPS-TCM gathered some experiences. They found that the adherence of patients will be higher under the care of nurses, while the outpatient will perform worse for some reasons such as forget, boredom of taking medicine, ignore the diseases, the disease is controlled temporarily, fear of the ADR, and distrust the medicine (Table 1).

In addition, other factors included the subjects' gender, age, occupation, marital status, education, income, disease types, race, religion, socioeconomic status, instructions about how to take medicine, course of the treatment, the severity of disease, cost and adverse reactions, anxiety of subjects, fear of life quality, social support (comes from family and friends), and experience and self-management ability.

Through the implementation of MISPS-TCM project, the team thought there are three factors that influence the adherence of subjects that mainly comes from the study protocol, the researchers, and the subjects itself.

### 2.1. Study Protocol

(1) The protocol presents complexity, taking medicine frequently, and large dose. In TCM clinical trials, patients were requested to take medicine in large dose and frequently for the small drug loadings. It is difficult for some subjects to accept that (2) the followup frequently influences the daily life and work; (3) more invasive tests (such as blood tests, gastroscopy, etc.) are difficult for subjects to accept; (4) subjects had mental fatigue when they take part in long time trials of TCM; (5) due to the particular character of TCM, the placebo of Chinese medicine can be discriminated easily because of the appearance and smell, and this can make the patient have poor adherence; (6) taste of part of Chinese medicine is terrible, and it is difficult to endure; (7) they have more adverse reactions.

### 2.2. The Researchers

(1) Researchers are unfamiliar with the protocol of study, and not sufficiently informed subjects about all responsibilities; (2) there is bad or indifferent attitude of researchers; (3) they cannot answer questions for patients satisfactory in time and be distrusted; (4) they cannot handle the issue properly when they meet with adverse events; (5) they are too busy to receive the visitor in time; (6) communication disorders between the doctor and patients for lack of communication skills or use of too much professional terms; (7) they transform the workers frequently.

### 2.3. The Subjects

(1) The understanding of disease is not enough and does not attach importance to the clinical research; (2) they mistrust the researchers or not satisfied with the researchers' attitude; (3) they are not satisfied with curative effect of the test drug; (4) they have fear of other problems followed with ADR; (5) they have poor memory and cannot take medicine or visit on time. (6) they take part in the trial just for gain the physical examination or free medicine. (7) there have a long distance between address of patient and hospital, and the traffic is inconvenient. (8) they are busy at work; (9) they think do not have to take medicine as the symptoms have disappeared; (10) they are influenced by others around subjects who are negative; (11) the patients are introverted, and shy away from interaction.

## 3. Measures to Improve Adherence

For the above factors, relevant measures should be taken during enrollment and treatment to improve adherence.

Currently, there are more approaches and strategies on adherence, for example, giving placebo during enrollment, considering study design and conduct from the perspective of participants, and promoting patients' understanding and support to research.

Based on our experiences from MISPS-TCM, measures will be summarized and discussed as follows.

### 3.1. Prevention Approaches before Enrollment

The prevention approaches before enrollment mainly focus on the study protocol and researchers.

#### 3.1.1. Study Protocol

Study protocol should emphasize the practicability. A good top-level design is half of success. Based on meeting trial demands, clinic visits and outcome measurements should be reduced. Endpoints were selected as outcomes such as a composite of cardiovascular death and nonfatal reinfarction and nonfatal stroke and the events of revascularization in MISPS-TCM, which avoided affecting daily work and life of subjects to a great extent.

In addition, when designing the trial, placebo in precursor can be taken in long term trials to exclude potential subjects with poor adherence [13].

#### 3.1.2. The Researchers

When study protocol is relatively fixed, the key points to improve adherence after enrollment is that the researchers inform subjects sufficiently and are willing to participate the study. In MISPS-TCM, before signing informed consent form, investigators made appointment with subjects and the conversation focused on the following issues: (1) further understanding potential subjects' medical history and determining whether it meet the inclusion and exclusion criteria, (2) explaining them the informed consent form in detail to ensure them voluntary participation, (3) knowing some information about subjects such as their education and family to confirm whether they have high adherence and can participate in the trial throughout.

In the progress of obtaining consent from subjects, attention should be paid to the following points. (1) Get subjects' trust. (2) Researchers who explain information on informed consent to subjects should have better ability of communication and skills and provide relevant information easy to understand and answer queries with good attitude. What is more, researchers should clearly tell subjects how to cooperate. Then subjects determine whether they can offer dedication in research. (3) Time to consider on participation should be enough. A readable “Patient information table” can be provided, which is helpful for potential subjects to discuss. Thereby the condition that subjects are enrolled due to thoughtless consideration can be avoided. (4) Explain the possible risks or inconvenience such as side effects or follow-up visit. It may increase the difficulty of recruitment; however, it can enhance adherence after enrollment owing to nonadherent subjects part who want to get paid. (5) It should be specified that all therapies possibly have side effects. Potential adverse reactions of treatment as well as controlled drugs should be informed ahead of time. Once adverse events occur, subjects can keep contact with research staff at any time.

### 3.2. Measures to Improve Adherence after Enrollment

**Figure 1:**
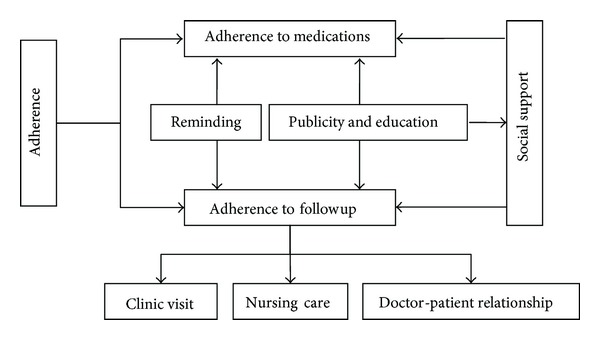
Measures to improve adherence after enrollment.

After participants sign informed consent form and are enrolled, researchers are recommended to strengthen communication with participants and guide them to rational drug use. Furthermore, with the help of research management office, participants should be offered a variety of convenient conditions if possible to avoid poor adherence. Relevant measures are addressed as Figure 1.

#### 3.2.1. Regular Reminding

*(a) Card for Medication, and Follow-Up Plans*. In MISPS-TCM, the card for reminding was placed in medicine box, including time, dose of medication, and visit. The card can be presented as a calendar, which was convenient for their family to see relevant information and provide assist.

*(b) Giving Out Drugs.* In MISPS-TCM, drugs were distributed to subjects according to how much would be taken in one follow-up period, and they were informed with the next clinic visit was informed. Drugs needed in the whole research should not be once given to subjects.

*(c) Sending Message or Telephone Call [14, 15]*. Researchers send message or telephone call on time of medications and clinic visit and request participants to provide feedback on time. Different contents can be sent to subjects. For the younger, the words can be lively and relaxing, and a joke or health tip can be added, while for the older, simple and kind message is appropriate instead of too professional expressions.

#### 3.2.2. Publicity and Education

The patient handbook was prepared in MISPS-TCM, and each participant can get one. Information related to this study was included such as secondary prevention of myocardial infarction, objective, method, composition of Chinese herbal medicine, and benefits and risks of participating in this trial. Thereby participants were able to fully understand issues associated with disease and our study. It is essential to further improve the subjects' adherence.

Family support for the adherence of patients plays an important role [16], so family and friends can be also involved in education and training on health care and clinical trials when necessary. Then they can have a correct knowledge of disease and trials.

Spreading knowledge of health care and research and emphasizing the importance of prescribed medications and followup will promote participant adherence. Also, it can form a better basis for future research.

Additionally, drugs not allowed to be taken during the trial can be listed in a card, to conveniently remind participants at any time.

#### 3.2.3. Social Support and Community Education [17]

Studies can involve family of participants and community, if possible, and make them realize the importance of adherence to researchers by adherence education. With the help of them, participants can be reminded to take medicine and visit on time. Understanding of family can also increase the confidence to complete the whole research.

#### 3.2.4. Follow-Up Plan

On the basis of following the study protocol, follow-up plan should depend on specific situation of participants such as avoiding the holiday or the inconvenient day. When the visit is over, researchers should remind patients of the next appointment time. For several absent participants, researchers should do telephone interviews in time and ask the reason.

#### 3.2.5. Relevant Service

Investigators and research management office provide convenience for participants during the duration of treatment and followup, for instance, offering traffic allowance, baby-sitting, or free parking and reducing waiting times. The reception room should be kept quiet and clean, and the temperature should be kept pleasant. Coffee, tea, magazines, newspapers, and television are available in this room.

Appropriate health-related information and concerns about their life not only can promote trust but can also confirm whether participants keep healthy.

#### 3.2.6. Doctor-Patient Relationship

Investigators and participants should increase open communication and trust each other. It has several benefits, including that (i) participants can get more information associated with health care in visits; (ii) when patients feel respected, they are willing to discuss some solutions with researchers; (iii) it is convenient to encourage participants to focus on disease changes or some possible adverse events. Also, the problems from participants should be handled in time, thereby removing the gap between doctors and patient. It makes sense to improve adherence.

Participants can contact the researchers by telephone or email to keep communication. It is necessary to assign the research staff responsible for follow-up visits of participants. The following situations must be avoided such as when researchers are busy with daily work, or the preset appointments are replaced, which will emerge as a great barrier to good communication and negotiation between participants and researchers.

#### 3.2.7. Other Measures

Providing rewards or compensation in several forms is also an effective measure including oral incentives, advising appropriate holiday, and providing transportation fee. Compensation should depend on trials itself and economic level in region where clinical trials are conducted. Moreover, sufficient attention should be paid to the principals that the reliability of trials is not changed as a result of compensation. Too low pay will affect the progress of enrollment to some extent, while too high can cause a false positive error.

Additionally, it is necessary to take reasonable solutions when poor adherence occurs in clinical trials. The concept of primary, secondary, and tertiary prevention approaches [18] has provided a good example for researchers committed to clinical trials of TCM.

## 4. Conclusion

Enhancing participant adherence is a crucial point and is also the emphasis and difficulty in clinical trials. When conducting clinical trials of TCM, appropriate adherence measures should be taken based on practically different situations. For instance, participants and investigators generally have high adherence in primary hospitals, so text message need not be sent. Additionally, cost and efficiency should be taken into consideration.

## Figures and Tables

**Table 1:** Table of factors related to participant compliance.

1	Forget
2	State of illness is controlled
3	Long course of treatment
4	High cost
5	Unblinding
6	Unwillingness of taking medicine
7	ADR
8	Complexity of instruction for taking medicine
9	Lack of correct understanding to disease
10	Lack of correct understanding to risk return
11	Ignore the disease
12	Low work satisfaction of doctor
13	Important event
14	Psychogeny
15	Improper follow-up plan
16	Difficulty in taking medicine
17	Unsure about treatment
18	Bad doctor-patient relationship
19	Difficult to care for some patients (the disabled, schizophrenic, et al.)
20	Asymptomatic disease
21	Complex description for medication
22	Difficult to get the medicine
23	Not familiar with the drug price
24	No reason
